# Supplementary material for: Sleep Deprivation Triggers Mitochondrial DNA Release in Microglia to Induce Neural Inflammation: Preventative Effect of Hydroxytyrosol Butyrate
Source: Antioxidants (Basel). 2024 Jul 12;13(7):833. doi: 10.3390/antiox13070833 (PMC11273532; doi:10.3390/antiox13070833)
Supplement: Supplementary file 1 [file antioxidants-13-00833-s001.zip › antioxidants-3058846-supplementary.pdf]

Supplementary Materials

Sleep Deprivation Triggers Mitochondrial DNA Release in Microglia to Induce Neural Inflammation: Preventative Effect of Hydroxytyrosol Butyrate

Yachong Hu [1], Yongyao Wang [1], Yifang Wang [1], Yuxia Zhang [1], Zhen Wang [1], Xiaohong Xu [2], Tinghua Zhang [2], Tiantian Zhang [1], Shuangxi Zhang [1], Ranrui Hu [1], Le Shi [1], Xudong Wang [1], Jin Li [3], Hui Shen [4], Jiankang Liu [1,5], Mami Noda [1,6], Yunhua Peng [1,*] and Jiangang Long [1,*]

1 Center for Mitochondrial Biology and Medicine, The Key Laboratory of Biomedical Information Engineering of Ministry of Education, School of Life Science and Technology, Xi'an Jiaotong University, Xi'an 710049, China; huyachong@stu.xjtu.edu.cn (Y.H.); wangyysep@foxmail.com (Y.W.); yifang@stu.xjtu.edu.cn (Y.W.); yx_zhang@stu.xjtu.edu.cn (Y.Z.); wz15761635541@stu.xjtu.edu.cn (Z.W.); sanjie1993@stu.xjtu.edu.cn (T.Z.); anneshuang@stu. xjtu.edu.cn (S.Z.); huranruiuse@stu.xjtu.edu.cn (R.H.); leshi@xjtu.edu.cn (L.S.); xudongwang@xjtu.edu.cn (X.W.); jkliu@uor.edu.cn (J.L.); maminoda39@gmail.com (M.N.)

2 School of Pharmacy, Chengdu Medical College, Chengdu 610500, China; xxh@cmc.edu.cn (X.X.); zhang_tinghua@cmc.edu.cn (T.Z.)

3 State Key Laboratory of Toxicology and Medical Countermeasures, Beijing Institute of Pharmacology and Toxicology, Beijing 100850, China; jinli9802@163.com

4 Department of Nutrition and Food Hygiene, Faculty of Naval Medicine, Naval Medical University, Shanghai 200433, China; shenhui@smmu.edu.cn

5 School of Health and Life Science, University of Health and Rehabilitation Sciences, Qingdao 266071, China

6 Research and Educational Resource Center for Immunophenotyping, RUDN University, 6 Miklukho-Maklaya St, Moscow 117198, Russia

* Correspondence: y.peng@mail.xjtu.edu.cn (Y.P.); jglong@mail.xjtu.edu.cn (J.L.)

This file includes:

Figures S1–S7
Tables S1

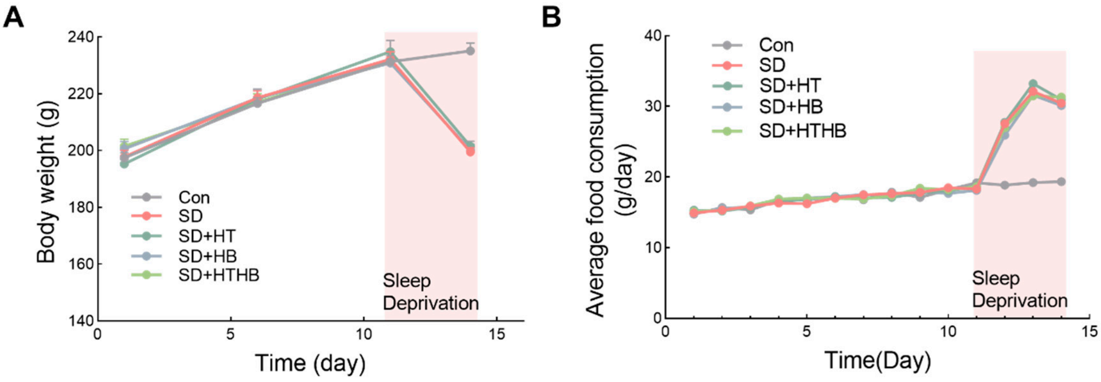

Figure S1. Changes in body weight and food consumption before and after the sleep deprivation. (**A**) Body weight, and (**B**) food consumption, in control (Con), sleep deprivation (SD), SD with hydroxytyrosol (HT), SD with b hydroxybutyric acid (HB), and SD with hydroxytyrosol butyrate (HTHB).

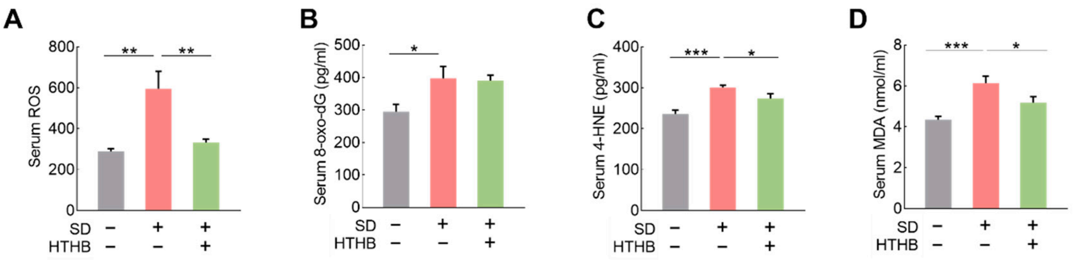

Figure S2. Sleep deprivation increases the oxidative stress in serum, which is partially attenuated by HTHB. (**A, B**) Serum levels of reactive oxygen species (ROS) (A) and 8-ox-dG (B). (**C, D**) The levels of 4-hydroxynonenal (4-HNE) (C) and malondialdehyde (MDA) (D) in the serum of rats. Values are mean ± SEM, n = 4, *p< 0.05, **p< 0.01, ***p< 0.001. The results were analyzed using one-way ANOVA.

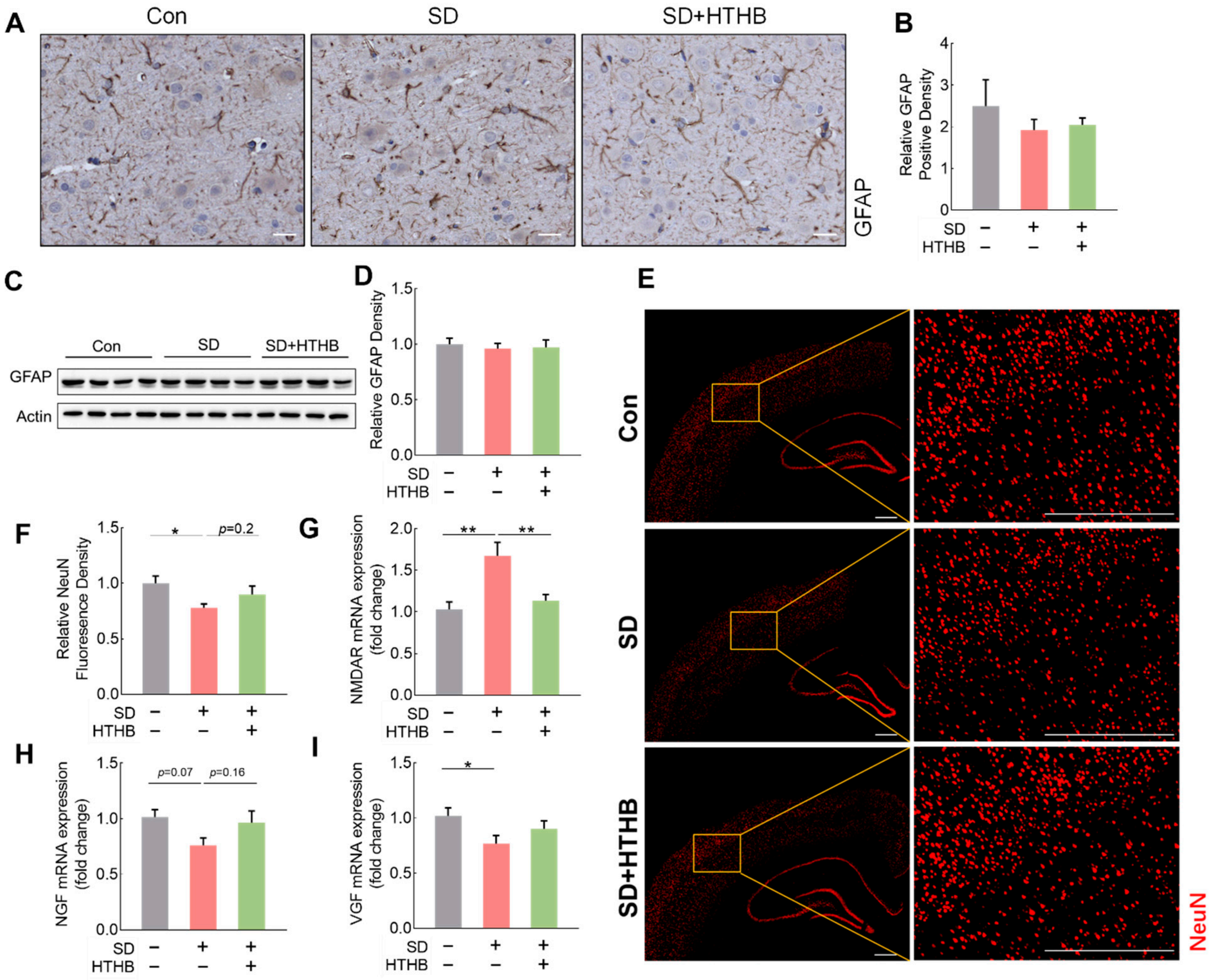

Figure S3. Sleep deprivation does not affect astrocytes but induces neuronal loss and affects neuronal function, which are alleviated by HTHB in the rat brain. (**A**) Immunohistochemistry of glial fibrillary acidic protein (GFAP), a marker of activated astrocyte, in control, after sleep deprivation (SD), and SD+HTHB in the cortex of rat brain. Scale = 25 μm, n = 4. (**B**) The relative GFAP-positive densities. (**C**) The typical example of Western blots of GFAP. (**D**) The relative GFAP density in control, SD, SD+HTHB in the rat brain, n = 8. **(E)** Immunofluorescent staining of NeuN (red) in rat brain. Scale = 500 μm, n = 4. **(F)** Relative immunofluorescent intensity of NeuN. (**G-I**) mRNA levels of N-methyl-D-aspartate receptor (NMDAR) (G), nerve growth factor (NGF) (H), and VGF nerve growth factor inducible (VGF) (I) in the rat cortex, n = 8. Values are mean ± SEM, n≥3, *p< 0.05, **p< 0.01, ***p< 0.001. The results were analyzed using one-way ANOVA.

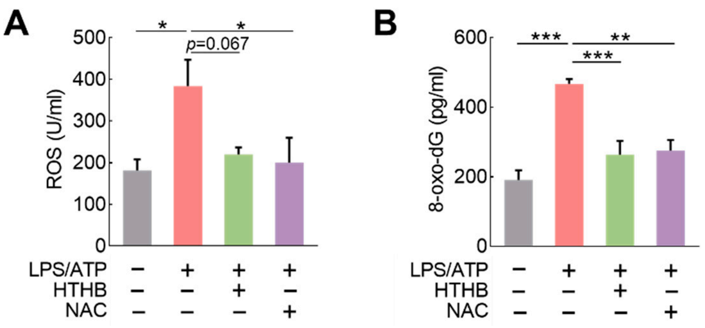

Figure S4. Effects of HTHB and N-acetyl cysteine (NAC) on LPS/ATP-induced ROS and 8-oxo-dG in N9 cells. (**A,B**) LPS/ATP treatment significantly increase the level of ROS (A) and 8-oxo-dG Levels (B), which are attenuated by both HTHB (10 µM) and NAC (0.5 mM). Values are mean ± SEM, n = 4, *p< 0.05, **p< 0.01, ***p< 0.001. The results were analyzed using one-way ANOVA.

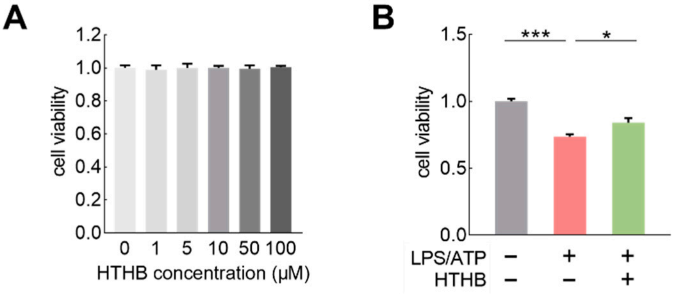

Figure S5. The effects of HTHB on N9 cell viability. **(A)** Treatment with HTHB at different concentrations did not affect the cell viability of N9 microglial cells after 48 h. **(B)** Treatment with HTHB (10 µM) for 48 h attenuated the LPS/ATP-induced cell death of N9 microglial cells. Values are mean ± SEM, n = 8, *p< 0.05, **p< 0.01, ***p< 0.001. The results were analyzed using two-way ANOVA or one-way ANOVA.

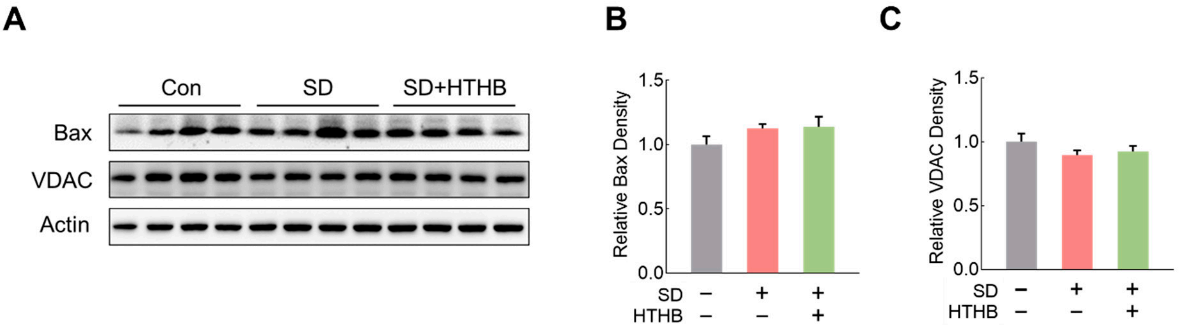

Figure S6. Expressions of Bax microchannel and voltage dependent anion channel (VDAC) were not affected by either sleep deprivation nor HTHB. (**A**) Typical example of Western blot of Bax and VDAC in the rat brain. (**B, C**) Relative expression densities of Bax (B) and VDAC (C). Values are mean ± SEM, n = 8, *p< 0.05, **p< 0.01, ***p< 0.001. The results were analyzed using one-way ANOVA.

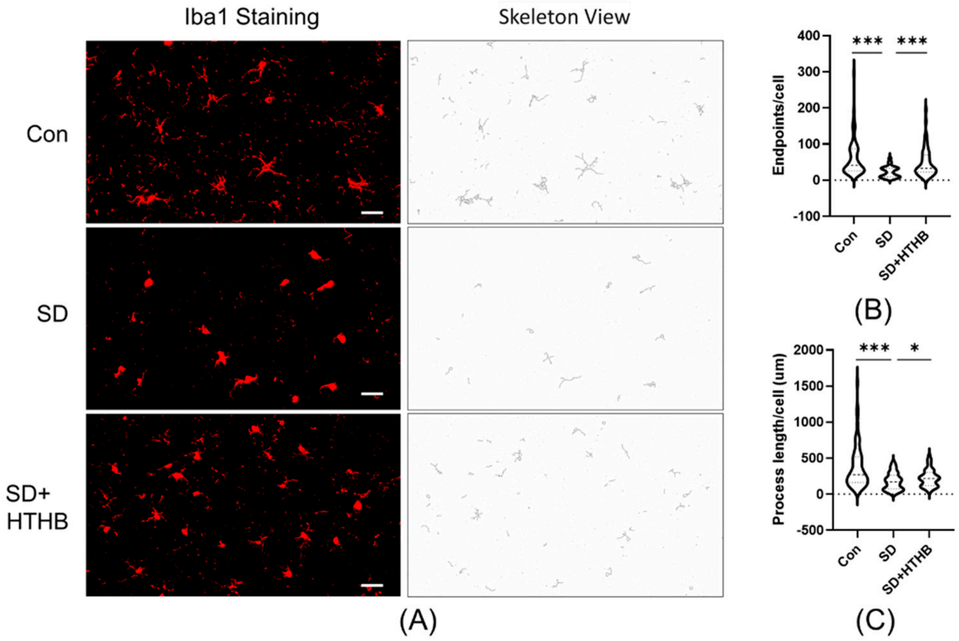

Figure S7. SD affects microglia morphology, which are ameliorated by HTHB. **(A)** Immunofluorescence staining of Iba1 and relative skeleton view. Scale bar = 20 μm. **(B)** Microglia endpoints per cell. **(C)** Process length per cell. Values are mean ± SEM, n = 30 (from 3 rat brain slices), $*p < 0.05$, $***p < 0.001$. The results were analyzed using one-way ANOVA.

Table S1. Primer list.

Species	Gene	Primer (5′-3′)
Rat	*β-actin*	Forward: AGATCAAGATCATTGCTCCTCCT<br>Revise: ACGCAGCTCAGTAACAGTCC
Rat	*NGF*	Forward: CATCGCTCTCCTTCACAGAGTT<br>Reverse: ATTACGCTATGCACCTCAGAGT
Rat	*NMDAR*	Forward: GAAAACCTCGACCAACTGTCC<br>Reverse: GTCGTCCTCGCTTGCAGA
Rat	*VGF*	Forward: CCCAGAACGAGGATTGCGAG<br>Reverse: CTGCCAGAGACTCCGAAGAG
Rat	*TNF*	Forward: ATGGGCTCCCTCTCATCAGT<br>Reverse: GCTTGGTGGTTTGCTACGAC
Rat	*IL6*	Forward: CTCTCCGCAAGAGACTTCCAG<br>Reverse: TGTGGGTGGTATCCTCTGTGA
Rat	*IL1β*	Forward: CAGCTTTCGACAGTGAGGAGA<br>Reverse: TTGTCGAGATGCTGCTGTGA
Rat	*18s*	Forward: CGAACGTCTGCCCTATCAACTT<br>Reverse: CTTGGATGTGGTAGCCGTTTCT
Rat	*D-loop*	Forward: AATCTACCATCCTCCGTG<br>Reverse: GACTAATGATTCTTCACCGT
Mouse	*β-actin*	Forward: GTTGGTTGGAGCAAACATC<br>Reverse: CTTATTTCATGGATACTTGGAATG
Mouse	*loop1*	Forward: AATCTACCATCCTCCGTGAAACC<br>Reverse: TCAGTTTAGCTACCCCCAAGTTTAA
Mouse	*loop2*	Forward: CCCTTCCCCATTTGGTCT<br>Reverse: TGGTTTCACGGAGGATGG
Mouse	*loop3*	Forward: TCCTCCGTGAAACCAACAA<br>Reverse: AGCGAGAAGAGGGGCATT
Mouse	*loop4*	Forward: AACGGATCCACAGCCGTA<br>Reverse: AGTCCTTCGGGCCATGATT
Mouse	*IL6*	Forward: TAGTCCTTCCTACCCCAATTTCC<br>Reverse: TTGGTCCTTAGCCACTCCTTC
Mouse	*TNFa*	Forward: CCCTCACACTCAGATCATCTTCT<br>Reverse: GCTACGACGTGGGCTACAG
